# Chimeric antigen receptor T cell therapies for multiple myeloma

**DOI:** 10.1186/s13045-019-0823-5

**Published:** 2019-11-21

**Authors:** Chao Wu, Lina Zhang, Qierra R. Brockman, Fenghuang Zhan, Lijuan Chen

**Affiliations:** 1 Department of Hematology, The First Affiliated Hospital of Nanjing Medical University, Jiangsu Province Hospital, Nanjing, 210029 China; 2 Department of Medicine, Division of Hematology, Oncology and Blood and Marrow Transplantation and Holden Comprehensive Cancer Center, University of Iowa, 585 Newton Rd., Iowa City, IA 52242 USA; 3 Molecular Medicine Program, University of Iowa, 585 Newton Rd., Iowa City, IA 52242 USA

**Keywords:** Chimeric antigen receptors, Multiple myeloma, Immunotherapy, Tumor immunology

## Abstract

Multiple myeloma (MM) is the second most common hematologic malignancy and remains incurable despite the advent of numerous new drugs such as proteasome inhibitors (PIs), immunomodulatory agents (IMiDs), and monoclonal antibodies. There is an unmet need to develop novel therapies for refractory/relapsed MM. In the past few years, chimeric antigen receptor (CAR)-modified T cell therapy for MM has shown promising efficacy in preclinical and clinical studies. Furthermore, the toxicities of CAR-T cell therapy are manageable. This article summarizes recent developments of CAR-T therapy in MM, focusing on promising targets, new technologies, and new research areas. Additionally, a comprehensive overview of antigen selection is presented along with preliminary results and future directions of CAR-T therapy development.

## Background

Multiple myeloma (MM) is a hematological malignancy characterized by the proliferation of transformed monoclonal plasma cells in the bone marrow (BM) [1]. MM is the second most common hematologic malignancy and is difficult to cure. It accounts for 1% of all cancers with a worldwide incidence rate estimated to be 6 –7 cases per 100,000 persons. Generally, MM patients show clinical symptoms including hypercalcemia, renal insufficiency, anemia, and bone destruction (CRAB) [2, 3].

Until the year 2000, the standard therapy for MM was melphalan- or doxorubicin-based regimens with corticosteroids. Introduction of proteasome inhibitors (PIs; e.g., bortezomib, carfilzomib, and ixazomib), histone deacetylase inhibitors (e.g., panobinostat), immunomodulatory agents (IMiDs; e.g., thalidomide, lenalidomide, and pomalidomide), and monoclonal antibodies (e.g., daratumumab and elotuzumab) has provided numerous therapeutic avenues for patients with MM. Despite these advanced therapies, most MM patients eventually relapse and become resistant to treatment, and the length and depth of response to therapies typically decrease in the following relapse. Therefore, it is essential to develop novel alternative treatment strategies that can combat chemotherapeutic resistance. Immunotherapy utilizing T cell immunity has become a new treatment to eliminate cancer cells. Chimeric antigen receptor (CAR) T cell therapy has emerged as a novel immunotherapy which modifies T cells with CAR, an artificial fusion protein that incorporates an extracellular antigen recognition domain, a transmembrane domain, and an intracellular domain including costimulation and signaling components [4, 5]. Many promising early results of CAR-T have been reported in MM, and more CAR-T clinical studies are currently being conducted [6–9]. This review summarizes the progress of CAR-T therapy in MM.

### Target selection for CAR-T therapy

The choice of target is critical for CAR-T therapy. Due to the genetic and phenotypic heterogeneity of MM cells, surface antigen expression on MM cells from the same patient might be variable [10–12]. Targeted antigens should be expressed specifically on MM cells to avoid on-target/off-tumor toxicity [13]. However, these MM-specific antigens have yet to be identified. Several antigens have been used as targets for CAR-T cell therapy against MM, including B cell maturation antigen (BCMA), CD19, CD138, signaling lymphocytic activation molecule 7 (SLAM7), and immunoglobulin light chains. The latest and most promising clinical trials are described in detail below.

### CAR-T therapy targeting BCMA

BCMA, a member of the tumor necrosis factor (TNF) superfamily, is exclusively expressed in a subpopulation of B cells, normal plasma cells, and malignant plasma cells. BCMA is not present in other hematological cells like hematopoietic stem cells or other tissues. It is closely associated with B cell-activating factor of the TNF family (BAFF) receptor, transmembrane activator, calcium modulator, and cyclophilin ligand interactor (TACI) [14]. Moreover, BCMA plays an essential role in regulating B cell maturation and differentiation into plasma cells. It facilitates B cell survival at different stages of development by engaging a proliferation-inducing ligand (APRIL) and BAFF [15]. Two main advantages of BCMA as an antigen for CAR-T therapy are the potential reduction of on-target/off-tumor toxicity and the lack of antigen-dependent reduction in CAR-T cell expansion [16]. A potential disadvantage of BCMA is that soluble BCMA can be released or shed from tumors into the the surrounding tissues and into the circulation. Soluble BCMA can potentially block the recognition of BCMA^+^ MM cells by BCMA-targeted CAR-T cells [17]. Nevertheless, the role of BCMA in the development of MM makes it the most popular target in MM CAR-T therapy.

In addition to CAR-T therapy, BCMA is also targeted for bispecific T cell treatment and antibody-drug conjugates (ADC). A bispecific T cell engager (BiTE) targeting BCMA and CD3ɛ (AMG420), and an anti-BCMA antibody-drug conjugate (GSK2857916) have demonstrated activity in the treatment of relapsed/refractory (RR) MM [18, 19].

### First-in-human CAR-T clinical trial targeting BCMA

The National Cancer Institute implemented the first clinical trial of CAR-T cells targeting BCMA (NCT02215967). The anti-BCMA CAR contains a murine single-chain variable fragment (scFv), a CD8α hinge and transmembrane region, a CD28 costimulatory domain, and a CD3ζ signaling domain. A total of 24 patients were enrolled, and the study tested 4 doses: 0.3 × 10^6^, 1 × 10^6^, 3 × 10^6^, and 9 × 10^6^ CAR-T cells/kg. The lowest dose of 0.3–3.0 × 10^6^ CAR-T cells/kg showed a minimal anti-tumor activity with an overall response rate (ORR) of 20%. Among the 16 patients treated with 9 × 10^6^ CAR-T cells/kg, the ORR was 81%. Notably, all the 11 patients that demonstrated a partial response or better were found to be minimal residual disease (MRD)-negative. However, significant cytokine release syndrome (CRS)-related toxicities were reported in patients treated at the highest dose (9 × 10^6^ CAR-T cells/kg). Additionally, the study suggested that patients with a high tumor burden were more likely to develop a high grade of CRS [8]. Currently, the development of many advanced BCMA-targeted CAR-T therapies is ongoing or completed, and most are registered as clinical trials for RRMM.

### bb2121

Data from the phase 1 study of bb2121 (NCT02658929) was published in the NEJM recently [20]. The BCMA-targeted CAR-T cell product bb2121 was infused in 33 RRMM patients. In this study, autologous T cells were transduced with a lentiviral vector encoding a novel CAR with an anti-BCMA scFv, a 4-1BB costimulatory domain, and a CD3ζ signaling domain [21]. Of these MM patients, 67% had an International Staging System (ISS) stage II or III disease, 27% had an extramedullary disease, and 45% had a high-risk cytogenetic profile defined by the presence of del(17p), t(4;14), or t(14;16). The study design included a dose-escalation phase and a dose-expansion phase. Doses of 50 × 10^6^, 150 × 10^6^, 450 × 10^6^, or 800 × 10^6^ CAR-T cells were infused in patients in the dose-escalation phase and 150 × 10^6^ to 450 × 10^6^ CAR-T cells in the dose-expansion phase. The ORR was 85% with 12 stringent complete responses (sCRs), 3 complete responses (CRs), 9 very good partial responses (VGPRs), and 4 partial responses (PRs). The median time to the first PR or better was 1 month, and the median duration of response was 10.9 months. The patients infused with at least 150 × 10^6^ CAR-T cells had rapid bone marrow (BM) clearance of plasma cells, and many patients achieved alleviation of extramedullary disease within 1 month. Of the 16 patients who obtained a PR or better, all achieved MRD-negative status (at ≤ 10^−4^sensitivity). CRS was reported in 25 patients (76%), and most events were grade 1 or 2. In addition, 14 patients (42%) had neurologic toxic effects including 1 patient with a reversible grade 4 neurologic toxic effect. The median time to onset of CRS was 2 days with a median duration time of 5 days. The persistence of CAR-T cells was examined at 1, 3, 6, and 12 months with 96%, 86%, 57%, and 20% of patients having detectable transgene levels, respectively [20, 22].

### bb21217

The phase 1 clinical trial of bb21217 is a next generation of anti-BCMA CAR-T therapy following bb2121. The structure of bb21217 is similar to bb2121 with the exception of the added phosphoinositide 3-kinase inhibitor bb007 during ex vivo culture. This modification was added to enrich the drug product for T cells displaying a memory-like phenotype and made CAR-T cells more persistent and potent. A multicenter phase 1 dose-escalation trial of bb21217 called CRB-402 (NCT03274219) recruited patients with RRMMs who had received ≥ 3 prior regimens. The planned dose levels were similar to CRB-401 (NCT02658929). Currently, 50 patients have been recruited and 7 patients have been treated with 150 × 10^6^ CAR-T cells and were evaluated at a 1-month time point. The results showed 1 sCR, 3 VGPRs, and 2 PRs. Three of 3 evaluable respondents obtained MRD-negative status. All 7 evaluable patients had a robust CAR-T cell expansion during the first 30 days. Five of the 7 patients experienced grade 1–3 CRS [23].

### LCAR-B38M

LCAR-B38M is a bispecific CAR-T cell product targeting 2 BCMA epitopes: VHH1 and VHH2. LEGEND-2, a single-arm, open-label, multicenter study (NCT03090659) evaluating LCAR-B38M in patients with advanced RRMMs, was conducted at 4 different sites in China. In the Second Affiliated Hospital of Xi’an Jiao Tong University, LCAR-B38M CAR-T cells (median CAR-T cell dose = 0.5 × 10^6^ cells/kg, [range, 0.07–2 × 10^6^]) were given in 3 infusions (20, 30, and 50% of total dose) in 57 patients. Of those, 37% had ISS stage III disease. Overall, the ORR was 88% (50 of 57), with 39 patients (68%) achieving CR, 3 patients (5%) achieving VGPR, and 8 patients (14%) achieving PR. All 39 patients with CR were MRD-negative assessed by 8-color flow cytometry. In addition, the median time to response was 1 month (range, 0.4 to 3.5). In total, 71% of MM patients had no detectable LCAR-B38M CAR-T cells in the peripheral blood at 4 months, and only 5 patients showed CAR-T cells at 10 months after infusion [9]. The safety results of the trial showed that the most common adverse events were pyrexia (91%), CRS (90%), thrombocytopenia (49%), and leukopenia (47%). CRS was mostly grades 1 and 2 (83%), and 4 MM cases (7%) had grade 3 events. The most common signs of end-organ injury in patients with CRS were liver functional abnormalities. The results from the other 3 sites have also been published (ChiCTR-ONH-17012285), with 17 patients infused with anti-BCMA CAR-T cells. All patients had BCMA-positive plasmablasts, 8 received auto-HSCT, and 5 had baseline extramedullary disease. High-risk cytogenetic abnormalities t(4,14) and del(17p) were reported in 6 patients by fluorescence in situ hybridization (FISH). The unfavorable prognosis markers gain (1q) and del (13q) were found in 11 and 6 patients, respectively. Two patients had a split FISH signal of IGH without known partner gene involvement. The doses of anti-BCMA CAR-T cells ranged from 0.21 to 1.52 × 10^6^ cells/kg. Eight patients were infused using 3 split doses, and 9 patients were administered with CAR-T cells as a single infusion. The ORR was 88.2% with 13 sCRs and 2 VGPRs at the first response evaluation 1 month after CAR-T cell infusion. Progression-free survival (PFS) rates were 82.4% at 6 months and 52.9% at 12 months, with 1-year overall survival (OS) rate being 82.3%. Sixteen patients experienced varying degrees of CRS, and 1 patient died due to tumor lysis syndrome in addition to CRS. Notably, in patients that responded, most were negative for BM MRD, but clonal plasma cells expressing BCMA always reappeared. No correlation was identified between disease relapse with age, gender, cytogenetic markers, conditioning scheme, CAR-T cell infusion dosage, and delivery method, and initial CR or VGPR. Extramedullary disease was a poor prognostic factor, and patients who previously had auto-HSCT were more likely to obtain a sustained response [24].

### P-BCMA-101

P-BCMA-101 is a novel CAR-T cell product using an anti-BCMA Centyrin™ fused to a CD3ζ/4-1BB signaling domain. Centyrins are fully humanized and have high binding affinities. A transposon system (piggyBAC), rather than a viral vector, was used to make them smaller, more stable, and potentially less immunogenic. A phase 1 clinical trial has being conducted in patients with RRMM to assess the safety and efficacy of P-BCMA-101 (NCT03288493). Twelve patients were infused with 48–430 × 10^6^ P-BCMA-101 CAR-T cells in three weight-based cohorts. Respondents in these nine MM patients who have had their first 2-week assessment included one sCR, one with non-secretory disease near CR of the patient’s plasmacytomas by PET/CT, one VGPR, and five PRs. Only one patient had grade 2 cytokine release syndrome [25, 26].

### JCARH125

JCARH125 is a BCMA-targeted CAR-T product, containing a lentiviral CAR construct with a fully humanized scFv, optimized spacer, 4-1BB costimulatory, and CD3ζ signaling domains. A multicenter phase 1/2 trial of JCARH125 called EVOLVE (NCT03430011) is ongoing and is recruiting patients with RRMMs. The first 2 dose levels were 50 and 150 × 10^6^ CAR-T cells. Modified toxicity probability interval 2 (mTPI-2) was used to determine the escalation of dose. Each dose level will be evaluated on at least 3 patients. Thus far, 19 MM patients were enrolled, and 13 of them were treated with JCARH125. Eight patients were eligible for the initial evaluation of early clinical response. All 8 patients showed evidence of objective response (≥ MR). Three patients have been confirmed to respond to 50 × 10^6^ CAR-T cells (2 sCRs, 1 PR), and the remaining patients have not been confirmed yet. Grade 1 or 2 CRS was observed in 6 of the 8 (75%) patients [27].

### CT053

A multicenter investigator-initiated clinical study has been designed to evaluate CT053 (NCT03915184), a novel autologous CAR-T therapeutic that is genetically modified T cells comprising an extracellular anti-BCMA human scFv, in RRMMs. In total, 16 patients were infused with CT053. Most enrolled patients were treated with a single dose of 1.5 × 10^8^ cells, except for 1 case that was infused with 0.5 × 10^8^ cells and another who received 1.8 × 10^8^ CAR-T cells. Among the 16 patients, 13 of them reached the assessment point and achieved 3 CRs, 6 VGPRs, and 4 PRs. Only 3 patients were observed with grade 1–3 CRS without any neurotoxicity and dose-limiting toxicities [28].

### MCARH171

A phase 1 dose-escalation trial of MCARH171 is ongoing to evaluate BCMA-targeted CAR-T cell product’s safety and efficacy on RRMM patients (NCT03070327). This anti-BCMA CAR contains a humanized scFv, a 4-1BB costimulatory domain, and a truncated epidermal growth factor receptor safety system. To date, 11 patients were infused with BCMA-targeted CAR-T cells following a standard 3 + 3 dose design. The mean doses of the 4 dosages were 72 × 10^6^, 137 × 10^6^, 475 × 10^6^, and 818 × 10^6^ viable CAR-T cells. The clinical responses of evaluable patients demonstrated that the ORR was 64%. Four (40%) and 2 (20%) patients were observed with grades 1–2 and grade 3 CRS, respectively. Notably, the extent and duration of clinical responses were all dose-dependent. The patients who received lower doses (72 × 10^6^,137 × 10^6^ CAR-T cells) had a lower peak peripheral blood expansion compared with those treated with higher doses (475 × 10^6^, 818 × 10^6^ CAR-T cells). Furthermore, 16.7% (1 of 6) of patients infused with lower doses had a clinical response lasting > 6 months, while 60% (3 of 5) of patients treated with higher doses had a clinical response lasting > 6 months [29].

### BRD015

BRD015 is also a BCMA-targeted CAR-T product containing a lentiviral CAR with a murine anti-BCMA scFv and CD28 costimulation domain. A phase 1 CAR-T trial (ChiCTR-OPC-16009113) using BRD015 has been conducted by the Tongji Hospital of Tongji Medical College, China. A total of 28 MM patients including 26 RRMMs, 1 plasma cell leukemia, and 1 POEMS were enrolled and treated with 5.4–25.0 × 10^6^ CAR-T cells/kg. Twenty-two MM patients were separated into 2 groups based on the BCMA expression on MM cells detected by flow cytometry. Of these, 16 (BCMA^+^ plasma cells ≥ 50%) and 6 were grouped into the high- and low-BCMA groups, respectively. The ORRs were 87% in the high-BCMA group (73% CR) and 100% in the low-BCMA group (33% CR or VGPR). In addition, clinical responses were positively correlated with peak blood CAR-T cell levels. A potential effect is also demonstrated in patients with the POEMS syndrome [30, 31]. However, the murine BCMA epitope of BRD015 leads to a serious defect that patients would be no longer sensitive to the reinfusion of CAR-T cells.

### CT103A

The novel BCMA-targeted CAR-T cell CT103A, which includes a fully humanized BCMA scFv, has been engineered. A single-center, phase 1 trial using CT103A was reported in 2019 EHA and ASCO meetings (ChiCTR1800018137) where nine patients were infused with 1–6 × 10^6^ cells/kg CAR-T cells. All patients achieved a clinical response within 14 days with an ORR of 100% (67% sCR/CR). The CRS was mild in the first two dosages while one patient treated with the highest dose demonstrated dose-limiting toxicity. Notably, three patients who relapsed after infusing BRD015 achieved two CRs and one VGPR following the CT103A therapy [32].

Other data of completed and ongoing BCMA-targeted CAR-T trials are listed in Table 1. Overall, all BCMA-targeted CAR-T therapies showed remarkable efficacy and safety profiles, suggesting that BCMA is a promising target in MM treatment [33–35].

**Table 1:** BCMA-targeted CAR-T clinical trials in multiple myeloma

Trial site/company	No. of patients	Costimulatory domain	Conditioning	Study design	Efficacy	Safety	Registration number/references
National Cancer Institute, phase 1	24	CD28	CP/Flu	0.3/1/3/9 × 10^6^ CAR+T cells/kg	ORR, 20% (2 of 10) with 1 VGPR, 1 PR (lower doses) ORR, 86% (12 of 14) with 1 sCR, 8 VGPRs, 3 PRs (highest dose)	CRS: Gr3/4, none (lower doses) CRS: Gr3/4, 38% pts (highest dose) CRES: Gr3/4, 19% pts (highest dose)	NCT02215967 [6, 8]
Multisite phase 1, Bluebird (bb2121)	33	4-1BB	CP/Flu	50/150/450/800 × 10^6^ CAR+T cells	ORR, 85% (28 of 33) with 12 sCRs, 3 CRs, 9 VGPRs, 4 PRs	CRS: Gr1/2, 70% pts; Gr3, 6% pts CRES: Gr1/2, 39% pts; Gr4, 3% pts	NCT02658929 [20]
Multisite phase 1, Bluebird (bb21217)	8	4-1BB	CP/Flu	150/450/800/1200 × 10^6^ CAR+T cells	ORR, 86%(6 of 7) with 1 sCR, 3 VGPRs, 2 PRs	CRS: Gr1/2, 50% pts; Gr3, 12.5% pts CRES: Gr4, 12.5% pts	NCT03274219 [23]
Multisite phase1/2, Nanjing Legend, China (LCAR-B38M)(XJTU)	57	4-1BB	CP	0.07–2.1 × 10^6^ CAR+T cells/kg	ORR, 88% (50 of 57) with 39 CRs, 3 VGPRs, 8 PRs	CRS: Gr1/2, 83% pts; Gr3/4, 7% pts CRES: Gr1, 1.8% pts	NCT03090659 [9]
Multisite phase1/2,Nanjing Legend, China (LCAR-B38M)(RJ, JS, CZ)	17	4-1BB	CP/Flu	0.21–1.52 × 10^6^ CAR+T cells/kg	ORR, 88.2% (15 of 17) with 13 sCRs, 2 VGPRs	Mild CRS, 10 pts; severe but manageable CRS, 6 pts; died of a very severe toxic reaction, 1 pt	ChiCTR-ONH-17012285 [24]
Multisite phase 1, Poseida (P-BCMA-101)	12	4-1BB	CP/Flu	48–430 × 10^6^ CAR+T cells	ORR, 89% (8 of 9 ) with 1 sCR,1 near CR, 1 VGPR, 5 PRs	CRS: Gr2, 1 pt	NCT03288493 [25, 26]
Multisite phase1/2, Juno (JCARH125)	19	4-1BB	CP/Flu	50/150 × 10^6^ CAR+T cells	ORR, 100% (8 of 8) with 1 PR, 2 sCRs 1 CR, 2 VGPRs, 1 PR, 1 MR	CRS: Gr1/2, 75% pts, CRES, 38% pts	NCT03430011 [27]
Multisite phase1, China (CT053)	16	4-1BB	CP/Flu	0.5/1.5/1.8 × 10^8^ CAR+T cells	ORR, 100% (13 of 13) with 3 CRs, 6 VGPRs, 4 PRs	CRS: only 3 pts 1 each for Gr1-3 CRES: none	NCT03915184 [28]
Celgene/MSKCC, phase 1 (MCARH171)	11	4-1BB	CP/Flu	72/137/475/818 × 10^6^ CAR+T cells	ORR, 64%	CRS: Gr1/2, 40% pts; Gr3, 20% pts CRES: Gr2, 10% pts	NCT03070327 [29]
Single site phase 1 (BRD015)	28	CD28	CP/Flu	5.4–25.0 × 10^6^CAR+T cells/kg	ORR: BCMA strong expression group, 87%; BCMA weak expression group, 100%	CRS: Gr3, 14% pts	ChiCTR-OPC-16009113 [30]
Single site phase 1 (CT103A)	9	4-1BB	CP/Flu	1/3/6 × 10^6^ CAR+T cells/kg	ORR, 100%(9 of 9) with 4 CRs, 1 VGPR, 4 PRs	Mild CRS (first two dose levels) DLT: *n* = 1 (highest dose)	ChiCTR1800018137 [31]
Single site phase 1 (CART-BCMA)	25	4-1BB	CP or none	1–50 × 10^7^ CAR+T cells	ORR, 48%(12 of 25) with 1 sCR, 1 CR, 5 VGPRs, 5 PRs	CRS:Gr3/4, 32% pts CRES, 12% pts	NCT02546167 [33]
Single site phase 1, HRAIN Biotechnology, China	17	4-1BB	CP/Flu	9 × 10^6^ CAR+T cells/kg	ORR, 79%(11 of 14) with 3 sCRs, 4 CRs, 2 VGPRs, 2 MRs	Mild CRS	NCT03093168 [34]
Multisite phase 1 in China	4	4-1BB	CP/Flu	5/10 × 10^6^ CAR+T cells/kg	ORR, 100% (4 of 4) with 1 CR, 3 PRs	All CRS under Gr3	NCT03661554 [35]
Soochow University, China (BCMA- and CD19-targeted CAR-T combination trial)	8	OX40, CD28	CP/Flu	1 × 10^7^/kg CD19-targeted CAR+T cells; 2.5–8.2 × 10^7^/kg BCMA-targeted CAR+T cells	ORR, 80% (4 of 5) with 1 sCR, 1 VGPR, 2 PRs	Mild CRS	NCT03196414 [36]
Soochow University, China (BCMA- and CD19- targeted CAR-T combination trial)	9	OX40, CD28	Bu-CP + ASCT	1 × 10^7^/kg CD19-targeted CAR+T cells; 2.5-8.2 × 10^7^/kg BCMA-targeted cells	ORR, 100% (9 of 9) with 3 CRs, 6 VGPRs	Mild CRS	NCT03455972 [37]
Affiliated Hospital of Xuzhou Medical University, China (BCMA- and CD19-targeted CAR-T combination trial)	21	4-1BB	CP/Flu	1 × 10^6^/kg both BCMA-and CD19-targeted CAR+T cells	ORR, 95% (20 of 21) with 9 sCRs, 3 CRs, 5 VGPRs, 3PRs	CRS: Gr1–2, 86% Gr3, 5%	ChiCTR-OIC-17011272 [42]

*Abbreviations*: *BCMA* B cell maturation antigen, *CAR* chimeric antigen receptor, *CRS* cytokine release syndrome, *CRES* cell related encephalopathy syndrome, *pts* patients, *Gr* grade, *VGPR* very good partial response, *SD* stable disease, *CR* complete response, *PR* partial response, *sCR* stringent complete response, *ORR* overall response rate, *MR* minimal response, *RRMM* relapsed/refractory multiple myeloma, *DLT* dose-limiting toxicity, *ASCT* autologous stem cell transplantation, *CP* cyclophosphamide, *Flu* fludarabine, *Bu* busulphan

### CAR-T therapy targeting CD19

CD19 belongs to the immunoglobulin superfamily and acts as a dominant signaling component of a multimolecular complex on the surface of mature B cells. It is present in many B cell malignancies such as acute lymphocytic leukemia (ALL) and chronic lymphocytic leukemia (CLL) [38]. CD19 is rarely expressed on MM cells, thus not an ideal target for the treatment of MM. However, recent studies have revealed that CD19 is expressed on a minor MM stem cell subset. The multiple myeloma stem cells (MMSCs) are defined as a population of tumor cells that possess the capabilities of self-renewal and drug resistance [39]. CD19 is also associated with the BM microenvironment-related drug resistance in MM [40]. Therefore, CD19 is a potential target for MM. Garfall et al. reported that the CD19-targeted CAR-T cell therapy (CTL019) infusion led to a durable complete response in an advanced refractory MM patient after a high-dose of melphalan treatment and autologous stem cell transplantation (ASCT) [7]. A further report from this group presented the complete data of the clinical trial (NCT02135406) including ten MM patients who were infused CTL019 cells after high-dose melphalan and ASCT. Two patients had significantly prolonged PFS after ASCT + CTL019 compared with ASCT alone, indicating that the CTL019 product and administration post-ASCT are safe and feasible in advanced MM patients [41].

### CD19- and BCMA-targeted CAR-T combination trial

In 2017, Fu et al. from the First Affiliated Hospital of Soochow University examined the safety and efficacy by combining CD19- and BCMA-targeted CAR-T cells in RRMM patients (NCT 03196414) [36]. The CAR used in this study was a third-generation construct containing an anti-BCMA and anti-CD19 scFv, a cytoplasmic portion of the OX40 and CD28 costimulatory moiety, and a CD3ζ T cell signaling domain. Eight RRMM patients received 1 × 10^7^/kg CD19-targeted CAR-T cells on day 0. Then, patients were infused with 40% BCMA-targeted CAR-T cells on day 1, and the remaining 60% cells were infused on day 2. Five of the 8 patients had the following response evaluation results: sCR (*n* = 1), VGPR (*n* = 1), PRs (*n* = 2), and SD (*n* = 1). CRS in all 5 treated patients was lower than grade 2 [36]. At ASH 2018, Fu et al. also presented results from a study of the CAR-T cell therapy (SZ-MM-CART02 study, NCT 03455972) [37]. The CAR-T cells were infused into patients on day 14 to day 20 after autologous transplantation. The dose and administration were the same as the first study. To date, 9 patients have been studied, and the ORR was 100% with 3 CRs, 2 VGPRs, and 4 PRs. This response improved to 3 CRs and 6 VGPRs after CAR-T therapy, and MRD negativity increased from 37.5 to 66.7% after CAR-T infusion and autologous transplantation. CRS in these patients were grades 1 and 2 [37].

Recently, the Affiliated Hospital of Xuzhou Medical University published the results of a single-arm, phase 2 trial (ChiCTR-OIC-17011272) targeting both BCMA and CD19 in patients with RRMM. Twenty-one patients were infused with both 1 × 10^6^ humanized anti-CD19 CAR-T cells/kg and 1 × 10^6^ murine anti-BCMA CAR-T cells/kg. All patients reached the evaluation point. The ORR was 95% with 9 (43%) sCRs, 3 (14%) CRs, 5 (24%) VGPRs, and 3 (14%) PRs. Seventeen (81%) evaluable patients had MRD while 94% of them achieved negative status within 1 month after CAR-T cell infusion. In addition, 19 (90%) patients experienced CRS with 86% grades 1–2 and 5% grade 3 [42]. Other data of CD19- and BCMA-targeted CAR-T combination trial are listed in Table 1.

### CAR-T therapy targeting NY-ESO-1

NY-ESO-1 belongs to the family of cancer/testis (CT) antigens. It is expressed in several types of cancers, including up to 60% of patients with relapsed MMs. Schuberth et al. constructed re-directed CD8þ effector T cells expressing CARs, which recognized the HLA-A*02-01-NY-ESO-1157-165 peptide complex [43]. This study showed that anti-NY-ESO-1 re-directed T cells could recognize MM cells endogenously expressing NY-ESO-1 and were able to lyse target cells and secrete antigen-specific Interferon (IFN)γ. Interestingly, some of the re-directed T cells showed an effector memory phenotype and secreted IFNγ when stimulated with NY-ESO-1 [43]. Therefore, NY-ESO-1 is another potential target for MM. Anti-NY-ESO-1 treatment has also been assessed in a phase 1/2 trial of TCR-transduced T cells in 20 MM patients after an autologous stem cell transplant. In total, 16 of 20 patients (80%) with advanced disease had good clinical responses, with a median PFS of 19.1 months [44]. In the latest study, the ORR at day 100 from 25 patients was 76% (1 sCR, 12 VGPRs, 6 PRs); at year 1, 13 patients were progression-free (52%), and 11 were responders (1 sCR, 1 CR, 8 VGPRs, 1 PRs) [45]. In addition, redirected CAR-T cells successfully exhibited anti-MM activity in an A2/NY-ESO-1157-specific manner [46].

### CAR-T therapy targeting Kappa light chain

It is recognized that T cell surface immunoglobulins are not generally expressed on plasma cells. However, Ramos et al. constructed a kappa-specific CAR that could recognize kappa-restricted MM cells [47]. Although cell-surface immunoglobulins are not expressed on all plasma cells, it was postulated that MM stem cells express surface immunoglobulins. Therefore, kappa light chain may also be a potential target for MM [47]. MDX-1097 is an anti-kappa free light chain mAb for MM. In a phase 1 clinical trial, 2 patients showed an encouraging result. One patient had a serum free light chain (FLC) level decreased by 55–61%. The other one showed an almost complete metabolic response determined by a PET scan 30 days after the infusion of MDX-1097 [48]. In a phase 2 multiple dose trial, 1 had a VGPR and 2 had PRs from 19 patients. Ten patients had SD six months after the initial infusion and 2 patients showed disease progression [48]. In another study by Ramos et al. using a CAR-targeting kappa light chain, 4 of 7 MM patients achieved responses after infusion with kappa-targeted CAR-T cells, including SD (> 24 months), minimal remission, or overall improvement of MM [47].

### CAR-T therapy targeting CD44 variant 6

CD44 is a glycoprotein which is broadly expressed on hematologic and epithelial tumors.CD44 isoform variant 6 (CD44v6) has been reported to be expressed by 43% of MM cases [49]. Bivatuzumab is a humanized monoclonal antibody targeting CD44v6 and was previously shown to be safe in a phase 1 radioimmunotherapy trial [50]. The main toxicity of bivatuzumab mertansine is against the skin, and the majority of skin reactions are reversible. However, one fatal drug-related adverse event was reported. Development was discontinued before reaching the maximum tolerated dose [50]. Anti-CD44v6 CAR-T cells were constructed and tested by Casucci et al. CD44v6-targeted CAR-T cells did not recognize hematopoietic stem cells and keratinocytes but did cause reversible monocytopenia [51]. The EURE-CAR-T project is to conduct a multicenter, first-in-man phase 1/2 clinical trial to demonstrate the safety and the efficacy of CD44v6-targeted CAR-T cell immunotherapy in acute myeloid leukemia and MM. The project started on January 1, 2017, and will continue until December 2020 (for more details, see https://www.eure-cart.eu/).

### CAR-T therapy targeting CD56

CD56 is a cell surface glycoprotein belonging to the immunoglobulin superfamily [52]. It is known to mediate cell-cell and cell-matrix interactions and is strongly expressed on malignant plasma cells in up to 78% of MM patients. It is also expressed on the surface of neural cells, epithelial cells, NK cells, and a subpopulation of activated T cells in normal tissues [52]. HuN901, a humanized monoclonal antibody which binds to CD56, showed a potent anti-myeloma activity in vitro and in vivo. Additionally, the study of HuN901 in murine models showed a well-tolerated dose [53]. These results support clinical trials for this agent. Lorvotuzumab mertansine (LM) is an antibody-drug conjugate which targets CD56^+^MM. The single-agent LM or LM in combination with lenalidomide and dexamethasone showed a promising activity against CD56^+^MM [54]. CAR-Ts, constructed by Benjamin et al., incorporating anti-CD56 scFv specifically reacted against MM cells in a preclinical study [55]. A serious concern with CD56-targeted CAR-T cells is the potential neurologic toxicity due to CD56 expression in the central and peripheral nervous systems.

### CAR-T therapy targeting CD70

CD70 (CD27L) is a member of the tumor necrosis factor family and is aberrantly expressed on some solid as well as hematological malignancies, including MM [56]. CD70 has highly restricted the expression on normal cells which makes it an attractive target for monoclonal antibody (mAb)-based therapies. SGN-70, a humanized anti-CD70 antibody developed by McEarchern et al., possesses Fc-dependent antibody effector functions and mediates anti-tumor activity in vivo [56]. BMS-936561 and SGN-75 are two specific monoclonal antibodies against CD70 [57, 58]. In a phase 1 study, an acceptable safety profile was reported. Results from a preclinical test also supported the safety and efficacy of a CD27-containing CAR targeting CD70-expressing tumors [59]. Furthermore, a lower risk of fratricidal killing is an advantage using CD70 antibody because CD70 is transiently expressed on immune cells. Two papers published in 2017 reported that CD70-targeted CAR-T therapies confer strong anti-tumor responses in human cancer cells and animal models [60, 61]. However, the therapeutic effect of CD70-targeted CAR-T cells in MM is not clear yet.

### CAR-T therapy targeting CD38

CD38 is a transmembrane glycoprotein involved in cell adhesion, signal transduction, and calcium regulation [62]. It is generally expressed on precursors of B cells, plasma cells, T cells, NK cells, and myeloid precursors. In normal tissues, it is also expressed on prostate cells, nervous system, guts, muscle cells, and osteoclasts [63]. CD38 is highly expressed in MM cells. Several monoclonal antibodies targeting CD38 have been tested clinically in MM. Daratumumab is the first human anti-CD38 monoclonal antibody approved to treat MM patients. It exerts anti-MM activity through antibody-dependent T cell-mediated cytotoxicity (ADCC), complement-dependent cytotoxicity, and antibody-dependent phagocytosis. Daratumumab is approved for the treatment of RRMM or as a single frontline agent or in a combination with other agents [64]. A second anti-CD38 mAb, SAR650984 (known as isatuximab), also showed potent preclinical and clinical anti-MM activity [65]. Given the anti-MM responses observed with daratumumab and isatuximab, the feasibility for developing CD38-targeted CAR-T cells is currently being explored. The results from the anti-CD38 mAbs suggested that anti-CD38 CAR-T cells could proliferate, produce cytokines, and lyse CD38^+^ MM cells. The potential problem is that these anti-CD38 CAR-T cells lyse not only CD38^+^ MM cells, but also CD38^+^ normal hematopoietic cells as well as other normal tissues expressing CD38. To avoid this problem, light-chain exchange technology is being used [66]. Additionally, a construction of anti-CD38 CAR-T with caspase-9-based suicide genes might be effective [67]. Recently, CD38-targeted CAR-T cells have been under investigation as a monotherapy for RRMM patients (NCT03464916). Many other clinical trials are exploring combinations of CD38-targeted CAR-T cells with other target antigens, including CD19 (NCT03125577) and BCMA (NCT03767751).

### CAR-T therapy targeting CD138

CD138, also known as syndecan 1, is a membrane protein and a member of the syndecan family of heparan sulfate proteoglycans. It is an adhesion molecule that binds to the extracellular matrix (ECM) molecules collagen and fibronectin, and also promotes cell proliferation [68, 69]. CD138 is expressed on most malignant and normal plasma cells but is absent from other hematopoietic cells, including T and B cells [70]. Therefore, CD138 is an ideal and specific target for the MM treatment. However, CD138 is also expressed in mature epithelial cells. Anti-CD138 could cause skin and/or mucosal toxicities (e.g., mucositis, stomatitis, hand/foot syndrome). Maytansinoid used the CD138 antibody (BT062, known clinically as indatuximab) as an immunoconjugate to treat MM patients. In a phase 1/2 clinical trial of BT062, only 1 out of 23 patients showed an objective clinical response [71]. However, when BT062 was combined with lenalidomide, the overall response rate was increased to 83% [72]. A study of CD138-targeted CAR-T therapy was conducted by Chinese PLA General Hospital (NCT01886976) on 5 RRMM patients, who were pretreated with chemotherapy and stem cell transplantation. The results showed that 4 of 5 patients had SD for more than 3 months, and 1 patient with advanced plasma cell leukemia had a reduction of MM cells in the peripheral blood from 10.5% to < 3% [73]. Despite the attractiveness of CD138 as a target for MM, the shedding of CD138 from malignant cells is a potential drawback. Avoiding skin toxicity and potential combination treatments are also topics of interest for future studies [74].

### CAR-T therapy targeting SLAMF7

SLAMF7 is a member of the signaling lymphocytic activation molecule family, which is under intense investigation as a target for immunotherapy in MM. It is also known as CD319 or CS1 [75, 76]. SLAMF7 is expressed on several hematologic cells such as plasma cells, NK cells, activated B cells and monocytes, some CD8^+^ T cells, and dendritic cells. SLAMF7 is absent on non-hematologic organs and hematopoietic stem cells, which makes it as a promising CAR target in MM [75, 77, 78]. The function of SLAMF7 in MM progression is still under investigation. Elotuzumab is a humanized immunoglobulin G kappa (IgG-κ) antibody which targets SLAMF7 [79]. Elotuzomab in combination with lenalidomide and dexamethasone was approved by the FDA in November 2015 for the treatment of MM patients who had received 1–3 prior therapies [79]. SLAMF7 expression on normal lymphocytes impacts the SLAMF7-targeted CAR-T cell therapy, especially in the culture and proliferation of SLAMF7-targeted CAR-T cells. Several SLAMF7-targeted CAR-T cell products are to be evaluated in clinical trials. UCARTCS1, which contained healthy and allogeneic T cells loaded with an anti-SLAMF7 CAR, was developed using TALEN-targeted gene editing [80]. When UCARTCS1 was tested in vitro and in mouse models, it showed the ability to target SLAMF7 and lyse MM cells.

### CAR-T therapy targeting GPRC5D

GPRC5D is a human orphan family C G protein-coupled receptor universally expressed in CD138^+^ cells [81]. Many studies have reported that GPRC5D is a promising target in MM treatment [82, 83]. However, studies of GPRC5D have only found GPRC5D mRNA expression in BM cells derived from MM patients. GPRC5D protein expression has been undetectable on MM cells via flow cytometry [84]. Recently, Smith et al. used quantitative immunofluorescence to detect the expression of GPRC5D on CD138^+^ cells and observed that GPRC5D is expressed on 98% of the CD138^+^ cells [85]. Specificity tests for the expression of GPRC5D in 30 non-plasma tissues showed that GPRC5D was only expressed on hair follicle cells. Based on this data, Smith et al. constructed GPRC5D-targeted CAR-T cells which exhibited significant anti-MM effects on MM cell lines and human MM cell lines (ffLuc+) xenografted in the NSG mice [85]. It is worth noting that GPRC5D-targeted CAR-T cells also showed activity in a murine model of post-BCMA-targeted CAR-T cells treated antigen escape. Overall, these results suggest that GPRC5D could play an important role in CAR-T therapy of MM patients.

### CAR-T therapy targeting NKG2DL

NKG2D is a highly conserved transmembrane protein that can recognize several ligands such as MICA, MICB, and the UL16-binding proteins (ULBP) which are upregulated in response to DNA damage, infection by certain pathogens, and malignant transformation. NKG2D ligands are expressed on many solid tumors and hematologic malignancies, including AML and MM. These ligands are usually absent on other normal tissues; therefore, NKG2DL is a novel promising target in MM CAR-T therapy. A single-center phase 1 study conducted by the Dana-Farber Cancer Institute has evaluated the safety and efficacy of NKG2DL-targeted CAR-T cells in RRMM patients. Five patients were infused with NKG2DL-targeted CAR-T cells following a modified Fibonacci “3 + 3” dose-escalation design. The study proposed to test four dosages ranging from 1 × 10^6^ to 3 × 10^7^ cells. Objective clinical responses to NKG2DL-targeted CAR-T cell therapy alone were not observed, and CRS was not reported in these five MM patients [86].

The detailed results of other targets besides a single BCMA target are summarized in Table 2.

**Table 2:** Non-BCMA-targeted CAR-T clinical trials in multiple myeloma

Trial site/company	Target	No. of patients	Costimulatory domain	Conditioning	Study design	Efficacy	Safety	Registration number/reference
University of Pennsylvania, Novartis	CD19	10	4-1BB	HDM + ASCT	5 × 10^7^ CAR+T cells	ORR, 20% (2 of 10)	Mild CRS	NCT02135406 [41]
Baylor University	Kappa LC	7	CD28	CP or none	1.7 × 10^7^–1.9 × 10^8^ CAR+T cells/m^2^	4 SD	Mild CRS	NCT00881920 [47]
General Hospital of PLA, China	CD138	5	CD28	PCD, CP, or VAD	22-90 × 10^7^ CAR+T cells	4 SD.	Mild CRS	NCT01886976 [73]
Single-center phase 1 study/Dana-Farber Cancer Institute	NKG2DL	5	Dap10	None	1–30 × 10^6^ CAR+T cells	ORR, 0% (0 of 5)	Mild CRS	NCT02203825 [86]

*Abbreviations*: *BCMA* B cell maturation antigen, *CAR* chimeric antigen receptor, *CRS* cytokine release syndrome, *ASCT* autologous stem cell transplantation, *HDM* high-dose melphalan, *CP* cyclophosphamide, *VAD* vincristine-doxorubicin-dexamethasone, *PCD* pomalidomide-cyclophosphamide-dexamethasone, *SD* stable disease, *ORR* overall response rate

## Future directions for CAR-T therapy in MM

The efficacy of using CAR-T therapy for MM treatment has been confirmed, and more investigators have turned their attention to optimizing therapeutic protocols. The main areas of focus are concerned with preventing CAR-T-associated side effects and increasing the efficiency of CAR-T therapy. It is well known that CRS and graft-versus-host disease (GvHD) are the most common side effects in CAR-T therapy. IL-6 receptor antagonist, tocilizumab; IL-1 blocker, anakinra; GM-CSF blocker, lenzilumab; and corticosteroids are often used to relieve the toxicity of CRS [87–89]. Moreover, novel ex vivo culturing of T cells such as T-Rapa cells that can reduce IFN releasing and new CAR structures containing suicide genes like caspase 9 are also currently being developed [90–94]. GvHD often happens during CAR-T therapy because many scFvs are murine-derived which might elicit a host immune response and limit the efficacy of the treatment. Currently, many CARs incorporating a human scFv have been generated to reduce the potential of immunogenicity [95].

Other remaining issues for further studies include improvement of the efficacy and durability of CAR-T cells and identification of optimal timing for CAR-T cell infusion. Many studies suggest that a combination with PD1 inhibitor may cause CAR-T cells re-expansion and anti-MM activity in the progression of MM patients following CAR-T cell infusion. Apegylated interleukin-10 (IL-10) called pegilodecakin can improve the effect of CAR-T cells in patients alone or in a combination with PD-1 inhibitor [96–99]. Cohen et al. have also reported that MM patients at an early stage and prior to exposure to multiple lines of therapy may have better outcomes in response to CAR-T therapy [100, 101]. Target selection, structural improvements, and combination therapies will have a priority in the future research of CAR-T therapy in MM.

## Conclusion

The development of CAR-T therapy in MM has become an attractive research topic in the past few years. Many studies of CAR-T therapy with different CAR constructs have shown high overall response and tolerable safety profiles in RRMM patients. Many BCMA-targeted CAR-T cell clinical trials have begun to register patients with MM who have failed all available therapies. CAR-T therapies targeting different antigens or in combination with different drugs are under preclinical and clinical studies. Future development and research on increasing the duration of responses, combining CAR-T therapy with different treatment modalities, and reducing potential toxicities will certainly help to further refine the role of CAR-T therapy in the management of MM.

## Data Availability

Data sharing is not applicable to this article as no datasets were generated or analyzed during the current study.

## Abbreviations

ADC: Antibody-drug conjugates
ADCC: Antibody-dependent cell-mediated cytotoxicity
AE: Adverse event
ALL: Acute lymphocytic leukemia
APRIL: A proliferation-inducing ligand
ASCO: American Society Clinical Oncology
ASCT: Autologous stem cell transplantation
ASH: American Society of Hematology
BAFF: B cell-activating factor of the TNF family
BCL2: B cell lymphoma-2
BCMA: B cell mature antigen
BsAb: Bispecific antibody
CARs: Chimeric antigen receptors
CAR-T: Chimeric antigen receptor-modified T cells
CD: Cluster of differentiation
CLL: Chronic lymphocytic leukemia
CR: Complete remission
CRAB: Hypercalcemia, renal insufficiency, anemia, and bone destruction
CRS: Cytokine release syndrome
DLBCL: Diffuse large B cell lymphoma
ECM: Extracellular matrix
EHA: European Hematology Association
FcRγ: Fc receptor γ chain
FDA: Food and Drug Administration
FISH: Fluorescence in situ hybridization
FLC: Free light chain
GM-CSF: Granulocyte-macrophage colony-stimulating factor
GvHD: Graft-versus-host disease
ICOS: Inducible costimulator
IFNγ: Interferon-γ
IL: Interleukin
LDC: Low-density lipoprotein
LM: Lorvotuzumab mertansine
mAb: Monoclonal antibody
MCL1: Myeloid cell leukemia-1
MHC: Major histocompatibility complex
MM: Multiple myeloma
MMSC: Multiple myeloma stem cell
MR: Minimal remission
MRD: Minimal residual disease
MTD: Maximum tolerated dose
mTPI-2: Modified toxicity probability interval 2
NK: Natural killer
NSG: NOD scid gamma
ORR: Overall response rate
PD: Progressive disease
PD-1: Programmed cell death protein 1
PET-CT: Positron emission tomography-computed tomography
PFS: Progression-free survival
PN: Peripheral neuropathy
PR: Partial remission
PR: Partial remission
qPCR: Quantitative polymerase chain reaction
RD: Lenalidomide and dexamethasone
RRMM: Relapse/refractory multiple myeloma
ScFv: Single-chain variable fragment
sCR: Strict complete remission
SD: Stable disease
SLAMF7: Signaling lymphocyte activation molecule F7
TAC: T cell antigen coupler
TACI: Transmembrane activator and CAML interactor
TCR: T cell receptor
TNF: Tumor necrosis factor
VGPR: Very good partial remission
